# Water, sanitation, and hygiene conditions and prevalence of intestinal parasitosis among primary school children in Dessie City, Ethiopia

**DOI:** 10.1371/journal.pone.0245463

**Published:** 2021-02-03

**Authors:** Awoke Aschale, Metadel Adane, Melaku Getachew, Kebede Faris, Daniel Gebretsadik, Tadesse Sisay, Reta Dewau, Muluken Genetu Chanie, Amare Muche, Aregash Abebayehu Zerga, Mistir Lingerew, Mesfin Gebrehiwot, Leykun Berhanu, Ayechew Ademas, Masresha Abebe, Gebremariam Ketema, Mengistie Yirsaw, Kassahun Bogale, Fanos Yeshanew Ayele, Mequannent Sharew Melaku, Erkihun Tadesse Amsalu, Gedamnesh Bitew, Awoke Keleb, Gete Berihun, Tarikuwa Natnael, Seada Hassen, Mohammed Yenuss, Mengesha Dagne, Alelgne Feleke, Helmut Kloos

**Affiliations:** 1 Hygiene and Environmental Health/Infection Prevention and Control Unit, Dessie Comprehensive Specialized Hospital, Dessie, Ethiopia; 2 Department of Environmental Health, College of Medicine and Health Sciences, Wollo University, Dessie, Ethiopia; 3 Department of Medical Laboratory Science, College of Medicine and Health Sciences, Wollo University, Dessie, Ethiopia; 4 Department of Epidemiology and Biostatistics, School of Public Health, College of Medicine and Health Sciences, Wollo University, Dessie, Ethiopia; 5 Department of Health Systems and Policy, School of Public Health, College of Medicine and Health Sciences, Wollo University, Dessie, Ethiopia; 6 Department of Nutrition, School of Public Health, College of Medicine and Health Sciences, Wollo University, Dessie, Ethiopia; 7 Department of Pharmacy, College of Medicine and Health Sciences, Wollo University, Dessie, Ethiopia; 8 Department of Health Informatics, Institute of Public Health, College of Medicine and Health Sciences, University of Gondar, Gondar, Ethiopia; 9 Department of Epidemiology and Biostatistics, University of California, San Francisco, San Francisco, California, United States of America; University of New South Wales, AUSTRALIA

## Abstract

**Background:**

Intestinal parasitosis is a major public health problem that affects the health of primary school children in low- and middle-income countries where water, sanitation, and hygiene (WASH) conditions are deficient. Since there is a paucity of information on the prevalence and associated factors of this problem among primary school children in Dessie City in Ethiopia, this study was designed to address these gaps.

**Methods:**

A school-based cross-sectional study was conducted among 407 stratified-sampled primary school children in five primary schools at Dessie City from April to June 2018. Data were collected using a pretested structured questionnaire, an observation checklist and laboratory analysis of stool samples. Stool specimen from each study participant was collected using clean, properly labeled and leak-proof stool cup. A portion of stool from each study participant collected sample was processed using saline wet mount technique and examined by microscope. The remaining specimens were preserved with 10% formalin and transported to Dessie Comprehensive Specialized Hospital laboratory to be processed by using formol-ether concentration technique. Then, slide smears were prepared from each processed stool specimen and finally, it was microscopically examined with 10x as well as 40x objectives for the presence or absence of intestinal parasites. Factors significantly associated with intestinal parasitosis were determined using binary logistic regression model at 95% CI (confidence interval). Thus, bivariate (COR [crude odds ratio]) and multivariable (AOR [adjusted odds ratio]) logistic regression analyses were carried out. From the multivariable analysis, variables having a *p*-value of less than 0.05 were declared as factors significantly associated with intestinal parasitosis among primary school children.

**Main findings:**

The overall prevalence of intestinal parasitosis was found to be 16.0% (95% CI: 12.5–19.4%), of these, 50.8% were positive for protozoa, 32.2% for helminth infections and 16.9% for double co-infections. *Entamoeba histolytica* was the most prevalent parasite (29.2%), followed by *Giardia lamblia* (21.5%), *Ascaris lumbricoides* (18.5%), *Hymenolepis nana (*9.2%) and *Enterobius vermicularis* (4.6%). Prevalence rates were similar among government (16.3%) and private (15.7%) school children. Water consumption was less than 5 liters per capita per day in 4 of the 5 schools. Thirty-eight (9.3%) of primary school students reported that they practiced open defecation. About two-thirds (285, 70.0%) said they always washed their hands after defecation. Mother’s education (illiterate) (AOR = 3.3; 95% CI: 1.20–9.37), father’s education (illiterate) (AOR = 3.9; 95% CI: 1.40–10.82), fathers who could read and write (AOR = 3.3; 95% CI: 1.25–7.86), handwashing before meal (sometimes) (AOR = 2.2; 95% CI: 1.11–4.17) and poor knowledge of WASH (AOR = 9.3; 95% CI: 2.17–16.70) were statistically associated with presence of intestinal parasitic infections.

**Conclusion:**

We concluded that the prevalence of intestinal parasitosis in the study area among Grades 4–8 primary school children had public health significance. Factors significantly associated with intestinal parasitosis among primary school children’s were illiterate mothers and fathers, irregular handwashing of children before meals, and poor knowledge of WASH. Health education to improve students’ WASH knowledge and mass deworming for parasites are recommended as preventive measures; and improvements to the quality of WASH facilities in primary schools are strongly recommended to support these measures.

## Introduction

Access to safe water and sanitation facilities is directly linked to the overall health of individuals and communities. Water, sanitation and hygiene (WASH) may prevent an estimated 8% of deaths and 10% of the disease burden in developing countries [1, 2]. Improved access to WASH can benefit children’s education by increasing school attendance and learning opportunities [3, 4]. The health impact of having sanitation facilities and services for the safe disposal of human urine and feces, maintenance of hygienic conditions through services such as solid waste/garbage collection and disposal, as well as the treatment and proper disposal of sewage waste water is considerable [5].

United Nations Children’s Fund (UNICEF) formulated the Three Star Approach for WASH in Schools to improve the health of students: wash hands with soap, have access to drinking water, and provide clean, gender-separated toilets at school every day [6]. Deficiencies in the WASH program are contributing to acquisition of intestinal diseases, including intestinal parasitosis [7].

Globally, intestinal parasites are highly prevalent, particularly in low-income regions [8]. Among the estimated 3.5 billion people with parasitosis worldwide, 450 million of them, mostly children, are ill as a result of these infections [9]. According to the World Health Organization (WHO) and UNICEF Joint Monitoring Program, 663 million people lacked improved drinking water sources and 2.4 billion lacked improved sanitation facilities world-wide in 2015. Unsafe and insufficient quantity of drinking water, inadequate sanitation, and unimproved hygiene account for 7% of the global burden of disease and 19% of child mortality worldwide [2].

In Ethiopia too, the high incidence of diarrhea, helminthiasis, and high mortality rates are associated with poor sanitation and water supply facilities. In Ethiopian schools, WASH is generally provided by governmental and nongovernmental organizations [10]. Furthermore, a study conducted in Senbete and Bete towns, North Shoa Zone found that the overall prevalence of intestinal infections was 52.3% [11], while a study in Tigray Regional State, reported a similar prevalence of 51.3% [12]. A systematic review and meta-analysis covering studies from 1996 to 2019 across the regional states in Ethiopia found rates from 48.0% to 53.0%, with the highest rates among preschool and school-age children [13].

A previous study among school children in Ethiopia indicated that the prevalence of intestinal parasitosis varied between those in private and government schools [14–16]. The overall prevalence of helminthic infections in private schools in Jimma Town was 20.9% and in government schools 53.5%. Soil-transmitted helminth infection rates peaked in the 11–15 year age group in both private schools (25.0%) and government schools (57.0%). Nearly two-thirds (64.5%) of the students had a single infection, 30.7% had double co-infections, and 4.8% had triple co-infections [14].

A study done in Dagi Primary School in Amhara Regional State found eight species of intestinal parasites, with an overall prevalence of 77.9%; the predominant parasite was hookworm (23.6%), followed by *Giardia lamblia* (22.8%), *Entamoeba histolytica* (21.6%) and *Strongyloides stercoralis* (1.5%) [15]. Similarly, a study among Debre Elias Primary School in Amhara Regional State, Ethiopia, reported an intestinal parasitosis rate in school children of 84.3%, with dual infections of 14.2% and high infection rates for hookworm (71.2%), *Entamoeba histolytica* (6.7%) and *Strongyloides stercoralis* (2.4%) [16]. Another study in two primary schools at Harbu town, south Wollo zone revealed the overall prevalence of intestinal parasitosis was 21.5% [17].

Factors significantly associated with intestinal parasitosis among school children varied considerable among schools in Ethiopia [11, 14–17]. A study in East Gojam Zone in Amhara Regional State revealed that the lack of available safe water supply, absence of shoes and low level of parents’ education were associated with intestinal parasitosis among primary schools children [16]. In another two studies of primary school children in Ethiopian towns, absence of latrines, absence of handwashing facilities, quality of environmental sanitation and type of latrine were significantly associated with intestinal parasitosis [15, 18]. Another study reported that intestinal parasitic infection was higher in children whose fathers were farmers [15].

These and additional studies of the prevalence of intestinal parasitosis and associated factors in pre-school and school-age children in Ethiopia [13, 17, 18–23] provide little information on WASH-related factors in parasitosis. For instance, a study in Dessie referral hospital about prevalence of intestinal parasitic infection among pre-school children found a prevalence of 15.5% [24], where this study did not provide information about WASH associated factors. Therefore, the objective of this study was to determine the prevalence of intestinal parasitosis and WASH-associated factors among children in Dessie City primary schools. Results may help to guide government and nongovernmental organizations in the control and prevention of intestinal parasitosis among primary schools children.

## Materials and methods

### Study setting

**Fig 1:**
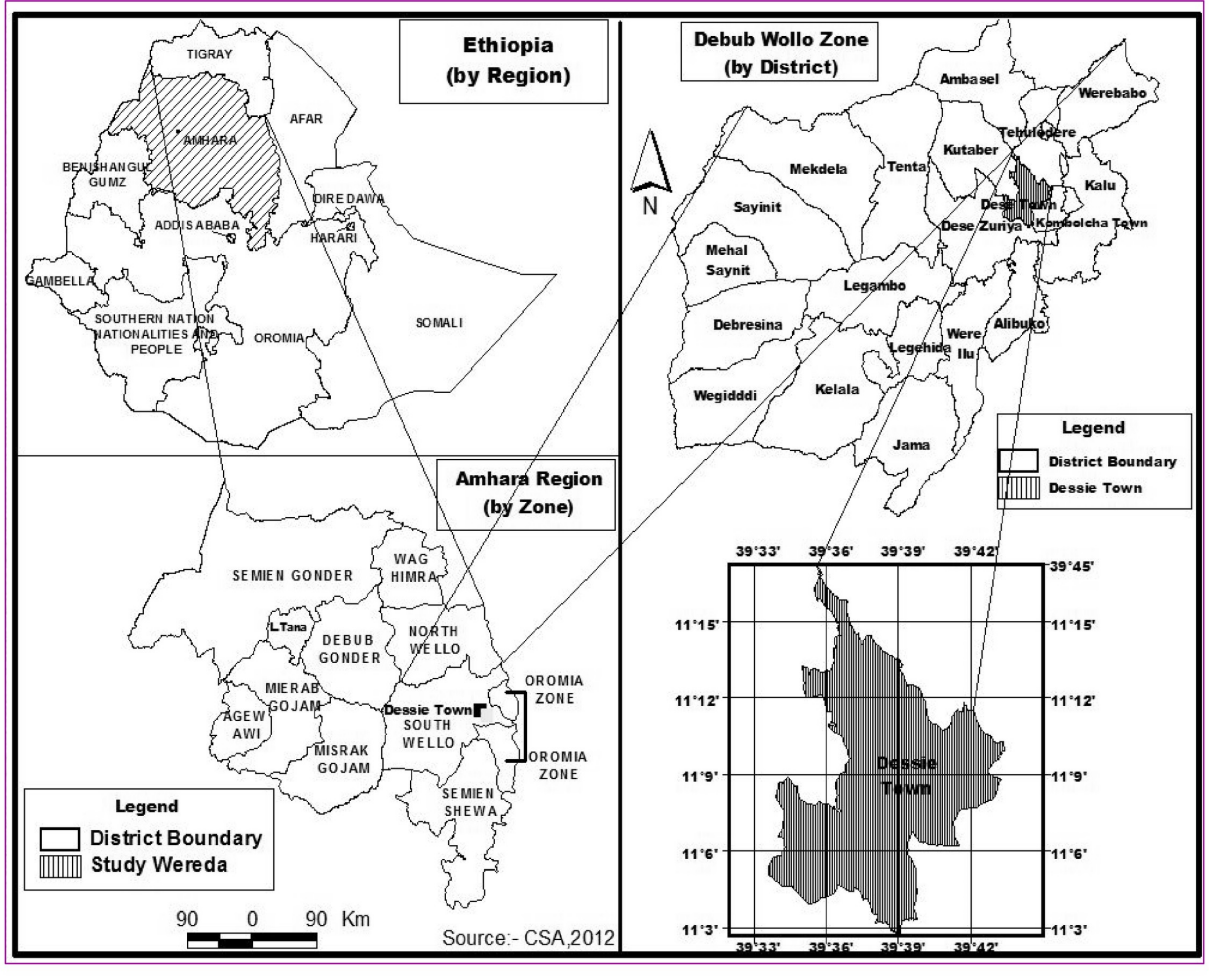
Map of the study area.

The study was conducted among Grade 4–8 students in five primary schools in Dessie City, Amhara Region, in the northern Ethiopian Highlands (Fig 1). There are 31 government and 14 private schools in Dessie, in which 26,457 students (13,358 male and 13,099 female) were enrolled in 2018. Among these, 16,663 students (8,495 male and 8,339 female) were in primary schools in Grades 4 to 8 [25].

### Study design and sample size

A school-based cross-sectional study was carried out from April to June 2018 using data collection methods of questionnaire survey, observation checklist and laboratory analysis of stool samples. The source population for this study was all students of the Grades 4–8 primary schools in Dessie City. Those who were night students or who had received anthelmintic drugs in the interval of two weeks prior to the actual specimen collection of the study were excluded. Sample size was estimated using the single population proportion formula with the following assumptions: 95% confidence level, 5% margin of error and reasonable estimate of proportion of intestinal parasitosis from other current studies 53.5% [14]. Then, the sample size became 384. After considering a 10% non-response rate, the final sample size was 423.

### Sampling technique

This study used a two-stage sampling method. During the first stage, five primary schools (three public and two private) that had Grades 4–8 were selected randomly out of 31 public and 14 private primary schools, from which sample size was proportionally allocated. During the second stage, stratified sampling methods were used to randomly select 33 sections (classes) out of a total 50 sections among the five schools; the sections were proportionally allocated to sample size. Then, study participants were proportionally allocated using the selected sections’ class lists. Finally, using each selected section’s attendance sheet as a sampling frame, study participants were selected using systematic random sampling technique.

### Stool specimen collection and laboratory examination

All study participants were instructed how to collect the stool samples. Then, stool specimen from each study participants were collected using properly labeled, clean, dry, leak proof, and wide-mouthed 5 ml plastic container with applicator stick. The collected stool specimen were processed immediately after collection. A portion of stool from each collected sample was processed using saline wet mount technique and examined by microscope. Then, the remaining specimens were preserved with 10% formalin and transported using a cooled box (kept at 2–8°C) to Dessie Comprehensive Specialized Hospital laboratory where they were processed and examined using formol-ether concentration techniques.

An estimated pea-size of stool was emulsified in 4 ml of 10% formol water. Next another 4 ml of 10% v/v formol water was added and mixed well by shaking. Four ml of diethyl ether was added after sieving of the emulsified stool. Then the tube was mixed for 1 min and immediately centrifuged at 750–1,000g for 1 min. After centrifuging, the parasites sedimented to the bottom of the tube and the stool debris collected in a layer between the ether and formol water. Then, slide smears were prepared from each processed stool specimen and examined using an Olympus microscope (using 10x and 40x objectives) for the presence or absence of intestinal parasites [26].

### Survey data collection and quality assurance

Data on socio-demographic, water, sanitation and hygiene parameters were collected using a structured questionnaire and on-the-spot-observation checklist. The tools were developed and translated from English to Amharic (local language) and re-translated from Amharic to English to ensure consistency. In order to increase the validity of the survey and to ensure that the students understood the questions, the questionnaire was pre-tested in a non-study primary school (Addis Alem Primary School) by taking 5% of the sample size (21 students) to check the validity of the questions; amendments were made accordingly.

Data collection was carried out by four data collectors, one supervisor, and the principal investigator. Data collectors and the supervisors were briefed about the aim of the study and trained for two days about data collection procedures, administration of the questionnaire and ethical issues. The principal investigator checked the data collection process on a daily basis. The interviews were carried out in a private setting to prevent social desirability bias. Before starting the interviews, students were oriented by the trained data collectors. In order to make the process convenient for respondents, all interviews were scheduled by appointment to provide times convenient for the respondents. Direct observation data for WASH facilities in the schools were collected by data collectors.

### Data management and analysis

The data were entered into the computer using EpiData software version 3.1 and then exported to SPSS software Version 20.0 for data cleaning and analysis. Descriptive analysis (frequencies, percentages) was used to describe the major characteristics of the study participants. Study participants who correctly answered a number of WASH knowledge questions equal to or above the mean were categorized as having good knowledge about WASH, whereas those who correctly answered a number of questions below the mean were classified as having poor knowledge about WASH. The prevalence of intestinal parasitic infections were determined using stool microscopy test result.

Multicollinearity of the independent variables was checked using the standard error (SE). Factors significantly associated with intestinal parasitosis were determined using binary logistic regression model at 95% CI (confidence interval). Thus, bivariate COR (crude odds ratio) and multivariable (AOR [adjusted odds ratio]) logistic regression analyses were carried out. Bivariate logistic regression analysis was done after dichotomizing the dependent variable by coding ‘1’ for positive and ‘0’ for negative stool results. From the bivariate analysis, variables with a *p-*value<0.25 were entered into the multivariable logistic regression analysis. From the adjusted analysis, variables having a *p*-value of less than 0.05 were declared as significantly associated with intestinal parasitosis. The model fitness was checked using the Hosmer-Lemeshow test and the *p-*value was > 0.05, indicating good fit [27].

### Ethical considerations

Ethical clearance for this study was obtained from the Ethical Review Committee of College of Medicine and Health Science, Wollo University. Parents or guardians of each study participant was signed an informed assent before data collection. After obtaining assent, all the information collected from the study participants was handled confidentially by omitting their names during the analysis and in presenting the results, and by using the data only for research purposes. To avoid ethical issues, the names of the five schools (both government and private) were not reported in this study. Stool analysis was done anonymously; in cases where stool samples showed parasitosis, we advised the student’s parent/guardian to obtain treatment for their child from a health facility.

## Results

### Characteristics of the study participants

Of the sampled 423 primary school children, 407 participated in an interview and provided stool samples (96.2% response rate). Three hundred thirty-nine (83.3%) students lived in urban areas and 68 (16.7%) in rural areas of Dessie City Administration. Three-quarters 306 (75.2%) of respondents were enrolled in public schools and 101 (24.8%) in private schools; 221 (54.3%) students were female and 186 (45.7%) were male. About two-thirds 309 (75.9%) of study participants were 10–12 years while mean age and standard deviation were 12.32±1.653 years. Regarding parents’ education, 71 (17.4%) of mothers and 67 (16.5%) of fathers were illiterate while 150 (36.9%) of mothers and 173 (42.5%) of fathers had attended secondary school or above (Table 1). With respect to knowledge of WASH, 117 (28.7%) (95%CI [24.4–33.0%]) of the study participants had poor knowledge, whereas 290 (71.3%) (95% CI [67.0–75.6%]) had good knowledge.

**Table 1:** Socio-demographic characteristics of Grades 4–8 primary school children, Dessie City, Ethiopia, April to June 2018.

Variables		Frequency (*n*)	Percentage (*%*)
School type	Public	306	75.2
	Private	101	24.8
Residence	Rural	68	16.7
	Urban	339	83.3
Sex	Male	186	45.7
	Female	221	54.3
Age (years)	10–12	309	75.9
	>12	98	24.1
Religion	Orthodox	213	52.3
	Muslim	170	41.8
	Protestant	24	5.9
Mother’s education	Illiterate	71	17.4
	Read and write*	77	18.9
	Primary	109	26.8
	Secondary or above	150	36.9
Father’s education	Illiterate	67	16.5
	Read and write*	86	21.1
	Primary	81	19.9
	Secondary or above	173	42.5
Mother’s occupation	Housewife	223	54.8
	Merchant	78	19.2
	Government employee	74	18.2
	Private employee	32	7.9
Father’s occupation	Farmer	55	13.5
	Merchant	123	30.2
	Government employee	154	37.8
	Private employee	75	18.4

*no formal education.

### Water, sanitation and hygiene conditions in the primary schools

#### Water supply condition

The sources of drinking water for the five primary schools included tap water, used by 339 (83.3%) students, and protected shallow-well water, used by 68 (16.7%) students. The sources of drinking water for students’ households included tap water supplied by the municipality after chlorine treatment for 339 (83.3%); protected shallow wells (29, 7.1%) and protected springs (39, 9.6%). Out of 5 schools, 3 practiced water disinfection treatment. Water consumption was less than 5 liters per capita per day in 4 of the 5 schools. Four schools had an alternative water source (back-up reservoir) to overcome interruptions in the main water supply (Table 2).

**Table 2:** Water, sanitation and hygiene conditions in primary schools in Dessie City, Ethiopia, April to June 2018.

Variables	Category	Frequency (*n*)	Percentage (*%*)
Water source for student’s household	Tap	339	83.3
	Protected dug well	29	7.1
	Protected spring	39	9.6
Water source for school	Tap	339	83.3
	Protected dug well	68	16.7
Water source maintenance at school	Sub-City Water and Sewerage Department	2	40.0
	School’s own plumber	2	40.0
	Community volunteers	1	20.0
Water treated by school	No	2	40.0
	Yes	3	60.0
Water use per capita per day (liters) in school	< 5	4	80.0
	≥5	1	20.0
School had alternative/back-up water source	No	1	20.0
	Yes	4	80.0
School’s water point was adequately maintained	No	1	20.0
	Yes	4	80.0
Place of defecation	Latrine	369	90.7
	Open defecation	38	9.4
Toilets provided privacy and security	No	207	50.9
	Yes	200	49.1
Toilets were hygienic and easy to use	No	374	91.9
	Yes	33	8.1
School had handwashing facility near toilets	No	4	80.0
	Yes	1	20.0
Path to toilet kept clean	No	2	40.0
	Yes	3	60.0
Number school children per toilet seat	≤ 50 boys	83	20.4
	≤ 25 girls	31	7.6
	>25 girls	189	46.4
	>50 boys	104	25.6
School had a health club	No	6	1.5
	Yes	401	98.5
Hygiene and sanitation part of health club	No	20	4.9
	Yes	387	95.1
Delegated body for hygiene promotion	No	41	10.1
	Yes	366	89.9
School sanitation facility designed to be easily and hygienically used	No	151	37.1
	Yes	256	62.9
Hygiene education provided by health club or teachers	No	38	9.3
	Yes	369	90.7
Hygiene maintenance participation	No	74	18.2
	Yes	333	81.8
Toilet and water point used correctly	No	226	55.5
	Yes	181	44.5
Student had been shown how to wash hands	No	330	81.1
	Yes	77	18.9
Handwashing after defecation	Sometimes	122	30.0
	Always	285	70.0

#### Sanitation condition

In regard to place of defecation, 38 (9.3%) of participants reported that they practiced open defecation. All toilets in both the governmental and private primary schools were pit latrines. In all primary schools, about half (49.1%) of the existing toilets did not provide privacy and security for the school children. Three hundred seventy-four (91.9%) of the participants reported that toilets were not hygienic to use. The proportion of toilet seats to the number of male students in the five school was 1:75, 1:38, 1:84, 1:28, and 1:30; the average ratio was one toilet seat for 51 male students (1:51). For female students, the ratios among the five schools were 1:84, 1:78, 1:108, 1:29 and 1:31; the average was one toilet seat for 61 female students (1:61) (Table 2).

#### Hygiene condition

Nearly all students (98.5%) reported that there was a health club in their school, and 96.5% of them stated that hygiene and sanitation were promoted by the health club. A majority 366 (89.9%) of the participants reported that there was a delegated body to promote hygiene in their school; 369 (90.7%) students had received hygiene education in school. One hundred eighty-one students (44.5%) said that they correctly used the toilets and water points and 334 (82.1%) of the students said that they always washed their hands before meals. About two-thirds (285, 70.0%) said that they always washed their hands after defecation (Table 2).

#### Prevalence of intestinal parasitosis

**Fig 2:**
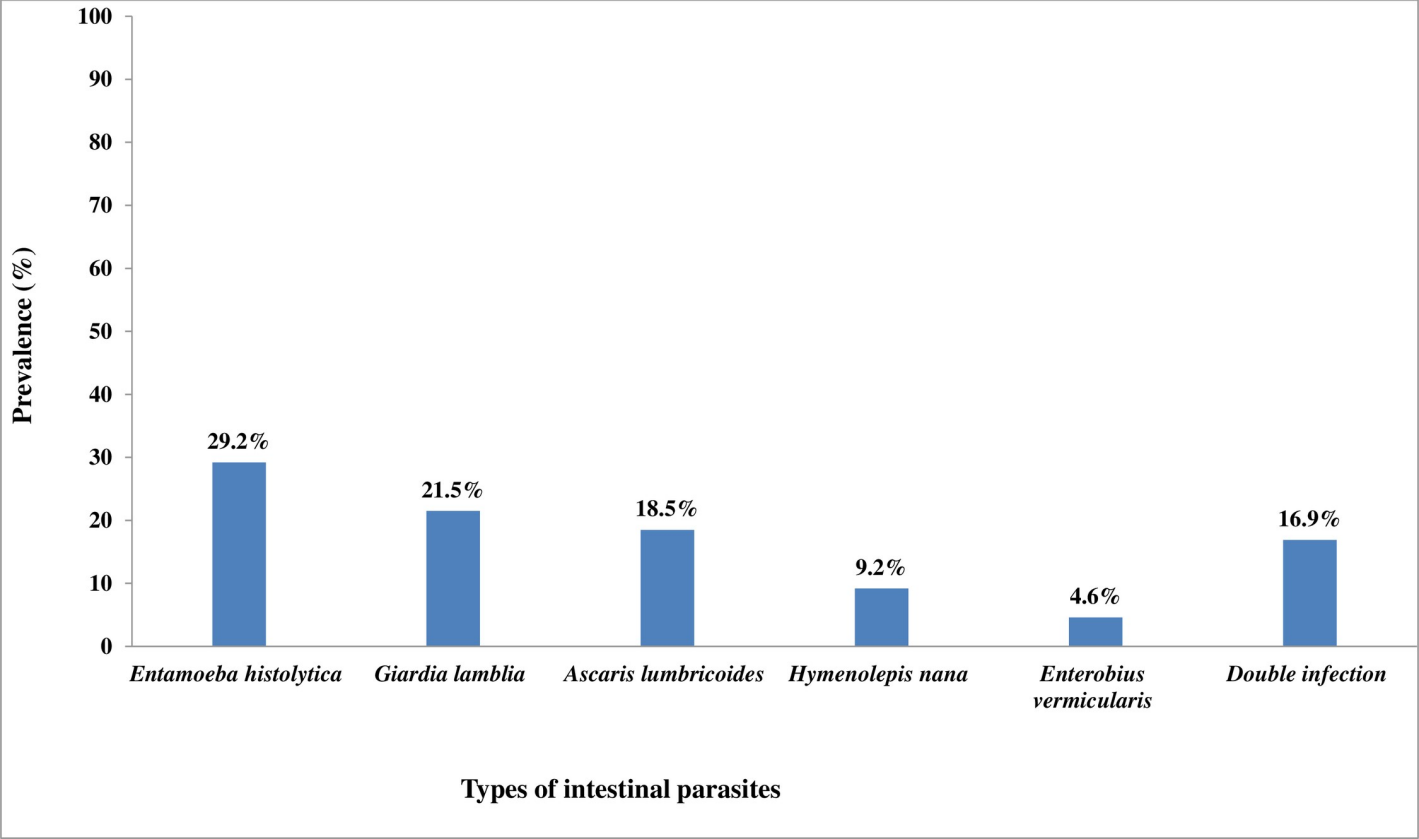
Types of intestinal parasitosis among Grades 4–8 primary school children in Dessie City, Ethiopia, April to June 2018.

Sixty-five of the 407 (16.0%) [95%CI: 12.5–19.4%] were positive for at least one intestinal parasites; of these, 33 (50.8%) were positive for protozoa, 21 (32.2%) for helminth infections and 11 (16.9%) for double infections. Prevalence rates were similar in government (16.3%) and private (15.7%) school children. Of those infected with intestinal parasites, *Entamoeba histolytica* was the most prevalent parasite (19, 29.2%), followed by *Giardia lamblia* (14, 21.5%), *Ascaris lumbricoides* (12, 18.5%), *Hymenolepis nana (*6, 9.2%) and *Enterobius vermicularis* (3, 4.6%) (Fig 2).

Of the 407 participants, 383 (94.1%) replied that they knew of intestinal parasitic diseases or were aware of amoebiasis; of these 103 (26.9%) knew of giardiasis, 143 (37.3%) knew helminthiasis, 17 (4.4%) amoebiasis and giardiasis, 12 (3.1%) amoebiasis, giardiasis and helminthiasis and 35 (9.1%) knew of other intestinal parasitic diseases (Table 3).

**Table 3:** Awareness of intestinal parasitic diseases among Grade 4–8 primary school students in Dessie City, Ethiopia, April to June 2018.

Parasitic diseases	Students who were aware (*n* = 383)	Percentage (*%*)
Amoebiasis	103	26.9
Giardiasis	73	19.1
Helminthiasis	143	37.3
Amoebiasis and giardiasis	17	4.4
Amoebiasis, giardiasis and helminthiasis	12	3.1
Others	35	9.1

### Factors associated with intestinal parasitosis

We found that factors significantly associated with intestinal parasitosis among primary school children were children who had illiterate mothers and fathers, sometimes washing hands before meals, and poor knowledge of WASH. Primary school children who had illiterate mothers were 3.3 times more likely to develop intestinal parasites (AOR = 3.3; 95% CI: 1.20–9.37) than those whose mothers had at least secondary school education. Primary school children who had illiterate fathers were 3.9 times more likely to develop intestinal parasites (AOR = 3.9; 95% CI: 1.40–10.82) than those whose fathers had at least secondary school education. Furthermore, we also found that primary school children who had fathers who could read and write were 3.3 times more likely to develop intestinal parasites (AOR = 3.3; 95% CI: 1.25–7.86) than those whose fathers had at least secondary school education (Table 4).

**Table 4:** Factors associated with intestinal parasitosis among primary school children from multivariable logistic regression analysis.

Variable	Category	COR (95%CI)	*p*-value	AOR (95%CI)	*p*-value
Mother’s education	Illiterate	4.9(2.22–11.06)	<0.001	3.3(1.20–9.37)	<0.001
	Can read and write*	3.5(1.58–8.10)	<0.003	1.2(0.43–3.46)	0.671
	Primary school	1.9(0.81–4.28)	0.783	1.3(0.51–3.49)	0.345
	Secondary school or above	Ref		Ref	
Father’s education	Illiterate	4.9(2.22–10.94)	<0.003	3.9(1.40–10.82)	0.012
	Can read and write*	3.8(1.75–8.27)	0.011	3.3(1.25–7.86)	0.001
	Primary school	2.6(1.11–5.91)	0.043	1.9(0.70–5.58)	0.542
	Secondary school or above	Ref		Ref	
Children’s handwashing before meals	Sometimes	1.9(1.07–3.31)	0.047	2.2(1.11–4.17)	0.037
	Always	Ref		Ref	
Children’s WASH knowledge	Poor	6.8(2.03–10.18)	0.001	9.3(2.17–16.70)	<0.001
	Good	Ref		Ref	

Ref, reference category; COR, crude odds ratio; AOR, adjusted odds ratio.

*no formal education.

Our findings also showed that primary school children who washed their hands irregularly (sometimes) before meals were 2.2 times (AOR = 2.2; 95% CI: 1.11–4.17) more likely to have intestinal parasites than those children who always washed their hands before meals. We also found that children who had poor knowledge of WASH were 9.3 times more likely to develop intestinal parasites (AOR = 9.3; 95% CI: 2.17–16.70) than those children who had good knowledge of WASH (Table 4).

## Discussion

This school-based cross-sectional study employed laboratory analysis of stool samples to examine the prevalence and types of intestinal parasitic infections among primary school children and a questionnaire survey, as well as observation to assess the WASH conditions in primary schools in Dessie City. We found that the overall prevalence of intestinal parasitosis was 16.0% (95% CI: 12.5–19.4%). Factors significantly associated with intestinal parasitosis infections were children who had illiterate mothers and fathers, children who had fathers who could read and write, irregular handwashing before meals and poor knowledge of WASH.

We found that the prevalence of intestinal parasitosis in Dessie City primary school children was 16.0%, a rate that was lower than that of similar studies conducted in southwestern Ethiopia (20.0%-76.7%) [28], Bahir Dar City primary schools (31.0%) [29], Wukro Town in eastern Tigray (60.7%), Ethiopia [30] and Senbete and Bete towns (52.3%), Ethiopia [11]. Our lower rate may be due to the fact that our study was conducted while a strong school WASH program was being implemented in Ethiopia, which in turn may have reduced the presence of intestinal parasitosis.

A current systematic review and meta-analysis study in Ethiopia showed that intestinal parasitosis prevalence among school and pre-school aged children was 48.0% and varied across geographical locations [13]. A study in Colombia indicated that gastrointestinal parasite infections in the tested population were mainly caused by suboptimal water quality with contamination by animal- and human-derived cysts [31]. Our findings are also in contrast with a 2016 study in West Africa, where reported high prevalence of intestinal parasitosis (64.7%) [32].

*Entamoeba histolytica* was the most prevalent parasite in our study (29.2%), which is consistent with a study conducted in Harbu Town, Ethiopia [17], whereas another study in children in central Albania found that *Entamoeba histolytica* was the least prevalent (0.3%) [33]. These discrepancies might be due to variations in school WASH programs between schools in our study and in Albania. However, the presence of double infection for parasite among school children was consistent with several studies in Ethiopia [14–16].

Our study showed that besides family education, only handwashing at critical times (before meals) and good WASH knowledge were significantly associated with intestinal parasitosis. However, a similar Ethiopian study conducted among Motta Town primary school children revealed several additional factors associated with intestinal parasitosis: residence, health education access, family education, shoe-wearing habits, handwashing practices, toilet availability and use, family income, availability of safe water, and open defecation practices were significant factors (*p* < 0.05) [20]. Only family education and handwashing practices were congruent with factors found in our study. A similar relationship was reported by a primary school study in the southern Ethiopian highlands [18]. This might be due to illiterate parents having less knowledge about intestinal parasites and having received less advice about prevention, and weak follow-up of their children’s WASH practices. Our findings were also consistent with a study among primary school children in Chencha Town, southern Ethiopia, which found that educational status of the household heads was associated with intestinal parasitosis [18].

Our findings of the direct relationship between intestinal parasitism and irregular handwashing before meals was stronger than that reported from another Ethiopian elementary school (AOR = 0.5, 95% CI [1.59–4.54]) [28] but similar to the results reported by a study in a school in Bahir Dar City (AOR = 2.33, CI [1.29–4.19]) [29]. The similarity of our findings with the Bahir Dar school children might be due to similar circumstances of the primary schools and the cities’ similar development status. Another study also found that handwashing with soap at key times reduced intestinal parasite re-infection among children [34], whereas open field defecation was significantly associated with intestinal parasitosis [35]. A similar study in Kenya associated handwashing after defecation at home with lower *S*. *mansoni* infection rates [36].

Participants with poor WASH knowledge were 9.3 times more likely to have intestinal parasitosis than participants with good WASH knowledge. This finding corroborates study results from a primary school in northeastern Ethiopia [21]. The finding may support the benefits of making improvements to school WASH activities. The results of our study illustrate that handwashing practices, parents’ awareness of intestinal parasitosis and school children’s knowledge of WASH may be important aspects of school WASH programs in collaboration with the health and education sectors in combating intestinal parasitosis.

Limitations exist in the present study. First, we collected self-reported data without conducting a detailed follow-up observational investigation of participants’ use of WASH facilities; self-reporting has been known to greatly overestimate real practice rates [37]. Second, this study was conducted in urban primary schools, which may limit conclusions and the generalizability of these findings to rural schools where WASH services may be less available. In spite of the above limitations, our study provides new insight into the prevalence of intestinal parasites in relation to WASH among primary school students in Dessie City; hence, our results might help in designing appropriate interventions for preventing intestinal parasitosis in the study area.

## Conclusions

Although this study found a lower prevalence rate (16%) than some other studies, it showed that intestinal parasite infection is a problem in Dessie City primary schools that requires public health attention in order to achieve the goal of zero infection level among children in the study primary schools. Factors significantly associated with intestinal parasitosis among primary school children were children’s who had illiterate mothers and fathers, children’s who had fathers who could read and write, irregular handwashing of children before meals, and poor knowledge of WASH. Therefore, stakeholders should give attention to preventing and controlling intestinal parasitosis by enhancing school WASH programs and maintaining the quality of WASH services that include handwashing facilities with water and soap availability. The programs should include reinforcing regular hand hygiene practices and improving WASH knowledge through continuing WASH education and advocacy.

## Supporting information

S1 File (DOCX)

S2 File (XLSX)

S3 File (DOCX)

## Abbreviations

AOR: adjusted odds ratio
CI: confidence interval
COR: crude odds ratio
UNICEF: United Nations Children’s Fund
WASH: water, sanitation and hygiene
WHO: World Health Organization

## References

[1] Fewtrell L, WHO. Water, sanitation and hygiene: Quantifying the health impact at national and local levels in countries with incomplete water supply and sanitation coverage. World Health Organization Environmental Burden of Disease Series No 15. 2007.

[2] Pruss-Ustun A, WHO. Safer water, better health: Costs, benefits and sustainability of interventions to protect and promote health. World Health Organization. 2008.

[3] En W, Gan G. Factors associated with use of improved water sources and sanitation among rural primary school children in Pursat Province, Cambodia. South East Asian J Trop Med. 2011;42(4):1022–31. 22299486

[4] Global Health Council. Water, sanitation and hygiene, global health berfing book: Global Health Council. 2013.

[5] Ryan EP. Baseline assessment of water, sanitation, and hygiene (WaSH) infrastructure and practices in government schools of the trapeang Chour Commune, Cambodia. J Environ Health Sci. 2017;3(1).

[6] Ryan E. Baseline Assessment of Water. Sanitation, and Hygiene (WaSH) Infrastructure and Practices in Government Schools of the Trapeang Chour Commune, Cam-bodia. 2017:1–8.

[7] Salem DAB, El-shazly A, Nabih N, El-Bayoumy Y, Saleh S. Evaluation of the efficacy of oral ivermectin in comparison with ivermectin–metronidazole combined therapy in the treatment of ocular and skin lesions of Demodex folliculorum. Int J Infect Dis. 2013;17(5):e343–e7. 10.1016/j.ijid.2012.11.022 23294870

[8] Barreto S, Miranda J, Figueroa J, Schmidt M, Munoz S, PP K-M, et al Epidemiology in Latin America and the Caribbean: Current situation and challenges. Int J Epidemiol. 2012;41(2):557–71. 10.1093/ije/dys017 22407860 PMC3324459

[9] Okyay P, Ertug S, Gultekin B, Onen O, Beser E. Intestinal parasites prevalence and related factors in school children, a Western City sample-Turkey. BMC Public Health. 2004;4(64). 10.1186/1471-2458-4-64 PMC544355 15615592

[10] Montgomery MA, Elimelech M. Water and sanitation in developing countries: including health in the equation. ACS Publications; 2007. 10.1021/es072435t 17265923

[11] Lewetegn M, Getachew M, Kebede T, Tadesse G, Asfaw T. Prevalence of intestinal parasites among preschool children and maternal KAP on prevention and control in Senbete and Bete Towns, North Shoa, Ethiopia. Int J Biomed Mater Res. 2019;7(1):1–7.

[12] Gebreyohanns A, Legese MH, Wolde M, Leta G, Tasew G. Prevalence of intestinal parasites versus knowledge, attitude and practices (KAPs) with special emphasis to Schistosoma mansoni among individuals who have river water contact in Addiremets town, Western Tigray, Ethiopia. PLoS ONE. 2018;13(9):e0204259 10.1371/journal.pone.0204259 30252865 PMC6155513

[13] Chelkeba L, Mekonnen Z, Alemu Y, Emana D. Epidemiology of intestinal parasitic infections in preschool and school-aged Ethiopian children: A systematic review and meta-analysis. BMC Public Health. 2020;20(117). 10.1186/s12889-020-8222-y 31992252 PMC6988312

[14] Debalke S, Worku A, Jahur N, Mekonnen Z. Soil transmitted helminths and associated factors among schoolchildren in government and private primary school in Jimma Town, Southwest Ethiopia. Ethiop J Health Sci. 2013;22(3):237–44. 10.4314/ejhs.v23i3.6 24307823 PMC3847533

[15] Alamir M, Awoke W, Feleke A. Intestinal parasites infection and associated factors among school children in Dagi primary school, Amhara National Regional State, Ethiopia. Health. 2013;5(10):1697–701.

[16] Workneh T, Esmael A, Ayichiluhm M. Prevalence of intestinal parasitic infections and associated factors among Debre Elias primary schools children, East Gojjam Zone, Amhara Region, North West Ethiopia. J Bacteriol Parasitol. 2014;15(1).

[17] Gebretsadik D, Tesfaye M, Adamu A, Zewde G. Prevalence of intestinal parasitic infection and its associated factors among school children in two primary schools in Harbu Town, North East Ethiopia: Cross-sectional study. Pediatr Health Med Ther. 2020;11:179–88. 10.2147/PHMT.S252061 32607051 PMC7297451

[18] Abossie A, Seid M. Assessment of the prevalence of intestinal parasitosis and associated risk factors among primary school children in Chencha town, Southern Ethiopia. BMC Public Health. 2014;14(166). 10.1186/1471-2458-14-166 24528627 PMC3933408

[19] Ansha MG, Kuti KA, Girma E. Prevalence of intestinal schistosomiasis and associated factors among school children in Wondo District, Ethiopia. J Trop Med 2020;2020 10.1155/2020/9813743 32280352 PMC7128065

[20] Asemahagn MA. Parasitic infection and associated factors among the primary school children in Motta town, Western Amhara, Ethiopia. Am J Public Health Res. 2014;2(6):248–54.

[21] Alyssa V, Nigusu A, Abera K, Yemane B, Michelle A. Knowledge, attitudes, and practices (KAP) of hygiene among school children in Angolela, Ethiopia Ethiop J Health Dev. 2010;51(2):73–9. PMC3075961 21155409

[22] Alemu M, Anley A, Tedla K. Magnitude of intestinal parasitosis and associated factors in rural school children, Northwest Ethiopia. Ethiop J Health Dev. 2019;29(1):923–28. 10.4314/ejhs.v29i1.14 PMC6341440 30700960

[23] Fentie T, Erqou S, Gedefaw M, Desta A. Epidemiology of human fascioliasis and intestinal parasitosis among school children in Lake Tana Basin, northwest Ethiopia. T Roy Soc Trop Med H. 2013;107(8):480–6. 10.1093/trstmh/trt056 23843557

[24] Gebretsadik D, Metaferia Y, Seid A, Fenta GM, Gedefie A. Prevalence of intestinal parasitic infection among children under 5 years of age at Dessie Referral Hospital: Cross sectional study. BMC Res Notes. 2018;11(771). 10.1186/s13104-018-3888-2 30373668 PMC6206668

[25] Dessie City Adminstration (DCA). Dessie town Education Department 2017/18 second quarter report, Dessie, Ethiopia. 2017/2018.

[26] Cheesbrough M. District laboratory practice in tropical countries: Cambridge University press 2006.

[27] Hosmer J, Lemeshow S, Sturdivant R. Applied logistic regression. 3rd ed. Hoboken, NJ: John Wiley and Sons 2013.

[28] Jejaw A, Zemene E, Alemu Y, Mengistie Z. High prevalence of Schistosoma mansoni and other intestinal parasites among elementary school children in Southwest Ethiopia: A cross-sectional study. BMC Public Health. 2015;15(600). 10.1186/s12889-015-1952-6 26135566 PMC4488975

[29] Hailegebriel T. Prevalence of intestinal parasitic infections and associated risk factors among students at dona Berber primary school, Bahir Dar, Ethiopia. BMC Infect Dis. 2017;17(362). 10.1186/s12879-017-2466-x 28535750 PMC5442677

[30] Kidane E, Menkir S, Kebede A, Desta M. Prevalence of intestinal parasitic infections and their associations with anthropometric measurements of school children in selected primary schools, Wukro Town, Eastern Tigray, Ethiopia. Int J Curr Microbiol Appl Sci. 2014;3(3):11–29.

[31] Hernández PC, Morales L, Chaparro-Olaya J, Sarmiento D, Jaramillo JF, Ordoñez GA, et al Intestinal parasitic infections and associated factors in children of three rural schools in Colombia. A cross-sectional study. PLoS ONE. 2019;14(7):e0218681 10.1371/journal.pone.0218681 31291262 PMC6619675

[32] Liao C-W, Fu C-J, Kao C-Y, Lee Y-L, Chen P-C, Chuang T-W, et al Prevalence of intestinal parasitic infections among school children in capital areas of the Democratic Republic of São Tomé and Príncipe, West Africa. Afr Health Sci. 2016;16(3):690–7. 10.4314/ahs.v16i3.8 27917201 PMC5112002

[33] Sejdini A, Mahmud R, Lim Y, Mahdy M, Sejdini F, Gjoni V, et al Intestinal parasitic infections among children in central Albania. Ann Trop Med Parastiol 2011;105(3):241–50. 10.1179/136485911X12987676649584 21801503 PMC4090787

[34] Mahmud MA, Spigt M, Bezabih AM, Dinant G-J, Velasco RB. Associations between intestinal parasitic infections, anaemia, and diarrhoea among school aged children, and the impact of hand-washing and nail clipping. BMC Res Notes. 2020;13(1). 10.1186/s13104-019-4871-2 PMC6941294 31898526

[35] Sitotaw B, Mekuriaw H, Damtie D. Prevalence of intestinal parasitic infections and associated risk factors among Jawi primary school children, Jawi town, north-west Ethiopia. BMC Infect Dis. 2019;19(341). 10.1186/s12879-019-3971-x 31023271 PMC6485161

[36] Njambi E, Magu D, Masaku J, Okoyo C, Njenga SM. Pintestinal parasitic infections and associated water, sanitation, and hygiene risk factors among school children in Mwea Irrigation Scheme, Kirinyaga County, Kenya. J Trop Med. 2020;2020. 10.1155/2020/3974156 PMC7238387 32454837

[37] Vedachalam S, MacDonald LH, Shiferaw S, Seme A, Schwab KJ, investigators P. Underreporting of high-risk water and sanitation practices undermines progress on global targets. PLoS ONE. 2017;12(5):e0176272 10.1371/journal.pone.0176272 28489904 PMC5425011

