# Supplementary material for: Water, sanitation, and hygiene conditions and prevalence of intestinal parasitosis among primary school children in Dessie City, Ethiopia
Source: PLoS One. 2021 Feb 3;16(2):e0245463. doi: 10.1371/journal.pone.0245463 (PMC7857601; doi:10.1371/journal.pone.0245463)
Supplement: S1 File — (DOCX) [file pone.0245463.s001.docx]

# Annex V. Amharic Questionnaire

የመጠይቁ መለያ ቁጥር___________

የትምህርት ቤቱ ስም_____________

የመኖሪያ ቀበሌ________________

መመሪያ አንድ- ከቀረቡት አማራጮች ውስጥ ተማሪው/ዋ የሚሰጡህ/ሽን ትክክለኛ መልስ ብቻ አክብብ/ቢ

ክፍል አንድ: ማህበራዊና ስነ-ህዝባዊ መረጃ

| ተ.ቁ | ጥያቄዎች | አማራጭ መልሶች | እለፍ |
| --- | --- | --- | --- |
| 101 | እድሜ | ________ ዓመት |  |
| 102 | የመኖሪያ አድራሻ | 1. ገጠር 2. ከተማ |  |
| 103 | ፆታ | 1. ወንድ 2. 2.ሴት |  |
| 104 | ሃይማኖት | 1. ክርስቲያን 2. እስላም 3. ፕሮስቴንታት 4. ሌሎች--------------- |  |
| 105 | የክፍል ደረጃ | ____________ |  |
| 106 | የወላጅ እናት የትምህርት ደረጃ | 1. ያልተማረ 2. ማንበብና መጻፍ 3. የመጀመሪያ ደረጃ 4. ሁለተኛ ደረጃና በላይ |  |
| 107 | የወላጅ እናት የጋብቻ ሁኔታ | 1. ያላገባች 2. ያገባች 3. ከባሏ የተለየች 4. የተፈታች 5. የሞተች |  |
| 108 | የወላጅ እናት ሥራ | 1. የቤት እመቤት 2. ነጋዴ 3. የመንግስ ሠራተኛ 4. የግል ተቀጣሪ 5. ሌሎች----------------- |  |
| 109 | የወላጅ አባት የትምህርት ደረጃ | 1. ያልተማረ 2. ማንበብና መጻፍ 3. የመጀመሪያ ደረጃ 4. ሁለተኛ ደረጃና በላይ |  |
| 110 | የወላጅ አባት ሥራ | 1. ገበሬ 2. ነጋዴ 3. የመንግስ ሠራተኛ 4. የግል ተቀጣሪ 5. ሌሎች_________ |  |
| 111 | አንተን/ችን ጨምሮ የቤተሰብ አባላት ብዛት በቁጥር ስንት ነው? | ________________ |  |

ክፍል ሁለት: ከውሐ አቅርቦት፣ ከአካባቢ ጽዳትና ከግል ንጽህና ጋር የተያያዙ መጠይቆች

| የውሐ አቅርቦትን በተመለከተ | | | | | | | | |
| --- | --- | --- | --- | --- | --- | --- | --- | --- |
| 201 | የመጠጥ ውሐ ምንጭ ከምን ነው (በቤተሰብ ደረጃ) | | | 1. ቧንቧ 2. የተጠበቀ ምንጭ 3. የተጠበቀ የጉድጓድ ውሐ 4. ሌሎች | | | |  |
| 202 | የመጠጥ ውሐ ምንጭ ከምን ነው (በት/ት/ቤት ደረጃ) | | | 1. ቧንቧ 2. የተጠበቀ ምንጭ 3. የተጠበቀ የጉድጓድ ውሐ 4. ሌሎች | | | |  |
| 203 | ያለውን የውሐ ምንጭ በኃላፊነት የሚጠግን ማን ነው | | | 1. የክፍለ ከተማ ውሐና ፍሳሽ 2. የት/ቤቱ ቧንቧ ሠራተኛ 3. ማኅበረሰብ | | | |  |
| 204 | በትምህርት ቤቱ ያለው ውሐ ይታከማል ወይ? | | | 1. አዎን 2. አይታከምም | | | |  |
| 205 | በትምህርት ቤቱ ውስጥ ያለው ውሐ የሚፈለገው መጠን | | | 1. ከ5 ሊትር በታች 2. ከ5 ሊትር በላይ | | | |  |
| 206 | ተለዋጭ የውሐ ምንጭ አለ ወይ? | | | 1. አዎን 2. የለውም | | | |  |
| 207 | በሁሉም ቦታ ለሁሉም ፍላጎት አገልግሎት የሚውል ውሐ አለ ወይ (ለመጠጥ፣ ለንጽህና፣ ወዘተ)? | | | 1. አዎን 2. የለውም | | | |  |
| 208 | ለአካል ጉዳተኞች አገልግሎት በሚውል መልኩ የተሰራ የውሐ አገልግሎት አለ ወይ? | | | 1. አዎን 2. የለውም | | | |  |
| 209 | በማንኛውም ጊዜ በትምህርት ቤቱ ውስጥ የውሐ አቅርቦት አለ ወይ? | | | 1. አዎን 2. የለውም | | | |  |
| 210 | ያሉ የውሐ አቅርቦት መስመሮች በሥርዓት እያገለገሉ እና እየተጠገኑ ነው ወይ? | | | 1. አዎን 2. የለውም | | | |  |
| ንጽህናን በተመለከተ | | | | | | | | |
| 211 | በትምህርት ቤቱ የጤና ክበብ አለ ወይ? | | | | 1. አዎን 2. የለውም | የለም ወደ 213 እለፍ | | |
| 212 | የተራ ቁጥር 211 መልስ አዎን ከሆነ የጤና ክበቡ የአካባቢ ፅዳትና የግል ንጽህናን የያዘ ነው ወይ? | | | | 1. አዎን 2. የለውም |  | | |
| 213 | ትምህርት ቤቱ ውስጥ ያሉ መምህራን ስለንጽህና ትምህርት እንዴት እንደሚሰጡ ሰልጥነዋል ወይ? | | | | 1. አዎን 2. የለውም |  | | |
| 214 | በትምህርት ቤቱ ውስጥ ስለንጽህና ትምህርት እንዲሰጥ የሚያበረታታ የተወከለ አካል አለ ወይ? | | | | 1. አዎን 2. የለውም |  | | |
| 215 | የትምህርት ቤቱ ፋሲሊቲዎች አሰራር በንጽህና ለመጠቀምና ለመጠገን ምቹ ነው ወይ? | | | | 1. አዎን 2. የለውም |  | | |
| 216 | በትምህርት ቤቱ ውስጥ የንጽህና ትምህርት በእውነቱ ይሰጣልን? | | | | 1. አዎን 2. የለውም |  | | |
| 217 | የትምህርት ቤቱ ልጆች/ተማሪዎች ንጽህናን ለመጠበቅ በንቃት ይሳተፋሉ? | | | | 1. አዎን 2. የለውም |  | | |
| 218 | ተማሪዎች መጸዳጃ ቤቶችን እና የውሐ ቧንቧዎችን እንዴት እንደሚጠቀሙ ያውቃሉ | | | | 1. አዎን 2. የለውም |  | | |
| 219 | ተማሪዎች እጃቸውን እንዴት መታጠብ እንደሚችሉ ያውቃሉ | | | | 1) አዎን   1. የለውም | የለም ወደ 221 | | |
| 220 | የ ተራ ቁጥር 219 አዎን ከሆነ ያሉ የእጅ አስተጣጠብ ሂደቶች ማሳየት ይችላሉ | | | | 1)ይችላሉ   1. አይችሉም |  | | |
| 221 | በማንኛውም ጊዜ በእጅ መታጠቢያ ቦታዎች ላይ ሳሙና ወይም እሱን የሚተካ አለ ወይ? | | | | 1. አዎን 2. የለውም |  | | |
| 222 | የትምህርት ቤቱ መምህራን የተማሪዎችን የንጽህና ፀባይ ለማሻሻል የሚጫዎቱት ሚና አለ ወይ? | | | | 1. አዎን 2. የለውም | የለም ወደ 224 እለፍ | | |
| 223 | የተራ ቁጥር 222 ‹‹አዎን›› ከሆነ የተማሪዎችን ንጽህና ለማሻሻል መምህራን ምን ይሰራሉ? | | | | 1. ንጽህናን በመጠበቅ 2. በመደበኛ ክትትል 3. ሽልማት በመስጠት 4. ሌላ ካለ---------- |  | | |
| 224 | የጥርስ ንጽህናህን/ሽን በመደበኛነት ሁልጊዜ ከምግብ በኋላ ትጠብቃለህ/ሽ? | | | | 1. አዎን 2. የለውም | የለም ወደ 226 እለፍ | | |
| 225 | ለ224 መልስዎ ‹‹ አዎን›› ከሆነ የትኛውን የጥርስ ንጽህና ዘዴ ትጠቀማለህ/ሽ? | | | | 1. ውሐ ብቻ 2. የወይራ 3. የጥርስ ሳሙና ከውሐ ጋር 4. ሌላ ካለ _____ |  | | |
| 226 | ምግብ ከመመገብ በፊት እጅ የመታጠብ ልምድ | | | | 1. ሁልጊዜ 2. አልፎ አልፎ |  | | |
| 227 | ከመጸዳጃ ቤት መልስ እጅህ/ሽ የመታጠብ ልምድ | | | | 1. ሁልጊዜ 2. አልፎ አልፎ |  | | |
| 228 | ህፃናትን ከተንከባከብህ/ሽ በኋላ እጅ የመታጠብ ልምድ | | | | 1. ሁልጊዜ 2. አልፎ አልፎ |  | | |
| 229 | ምግብ ከማዘጋጀትህ/ሽ በፊት እጅ የመታጠብ ልምድ | | | | 1. ሁልጊዜ 2. አልፎ አልፎ |  | | |
| 230 | ለማዳ አጫዋች እንስሳ እንደ ድመት በእጅ ከነኩ በኋላ የመታጠብ ልምድ | | | | 1. ሁልጊዜ 2. አልፎ አልፎ |  | | |
| 231 | ምግብ ከበላህ/ሽ በኋላ እጅ የመታጠብ ልምድ | | | | 1. ሁልጊዜ 2. አልፎ አልፎ |  | | |
| 232 | የተማሪው እጅና ጥፍር ላይ የሚታይ ቆሻሻ አለ ወይ? | | | | 1. አለ 2. የለም |  | | |
| የአካባቢ ጽዳት | | | | | | | | |
| 233 | | የምትጸዳዳው/ጂው የት ነው | 1. መጸዳጃ ቤት 2. ማንኛውም ቦታ 3. ውሐማ ቦታዎች አካባቢ | | | |  | |
| 234 | | በትምህርት ቤቱ ያለው መጸዳጃ ቤት ዓይነት? (መጸዳጃ ቤቱን መመልከት) | 1. ፒት ላትሪን/የተለምዶ 2. ቪአይፒ ላትሪን 3. ዋተር ፍላሽ | | | |  | |
| 235 | | በትምህርት ቤቱ ያለው መጸዳጃ ቤት በቂ መቀመጫ አለው ወይ? (በምልከታ) | 1. 1 መቀመጫ ለ25 ወንድ ተማሪዎችና በታች 2. 1 መቀመጫ ለ50 ሴት ተማሪዎችና በታች 3. 1 መቀመጫ ከ25 በላይ ሴት ተማሪዎች 4. 1 መቀመጫ ከ50 በላይ ወንድ ተማሪዎች | | | |  | |
| 236 | | ለሴትና ለወንድ ተማሪዎች የተለየ መጸዳጃ ቤት አለ ወይ? (ተማሪዎችን መጠየቅና መመልከት) | 1. አዎን 2. አይደለም | | | |  | |
| 237 | | መጸዳጃ ቤቶች ብቸኝነትንና ዋስትናን የጠበቁ ናቸው ወይ? (በምልከታ) | 1. አዎን 2. አይደለም | | | |  | |
| 238 | | መጸዳጃ ቤቶቹ ለአካል ጉዳተኞች ተገቢና በቀላሉ የሚደረሱ ናቸው ወይ? (በምልከታ) | 1. አዎን 2. አይደለም | | | |  | |
| 239 | | ለወንድ አካል ጉዳተኛ ተማሪዎቸ አንድ እንዲሁም ለሴት አካል ጉዳተኛ ተማሪዎቸ አንድ መጸዳጃ ቤት አለ ወይ ? (በምልከታ) | 1. አዎን 2. አይደለም | | | |  | |
| 240 | | መጸዳጃ ቤቶቹ ለመጠቀምና ንጽህናቸውን ለመጠበቅ ምቹ ናቸው ወይ? (በምልከታ) | 1. አዎን 2. አይደለም | | | |  | |
| 241 | | ከመጸዳጃ ቤቶቹ አጠገብ የእጅ መታጠቢያ አለ ወይ? (በምልከታ) | 1. አዎን 2. የለም | | | |  | |
| 242 | | መጸዳጃ ቤቶቹን ለማጽዳትና ለመጠገን እቅድ አለ ወይ? (እቅዱን በማየት) | 1. አዎን 2. የለም | | | |  | |
| 243 | | ወደ መጸዳጃ ቤቶች የሚወስዱ መንገዶች ምቹ ናቸው ወይ? (በምልከታ) | 1. አዎን 2. የለም | | | |  | |
| 244 | | መጸዳጃ ቤቶቹ በንጽህና እና በሥርዓት እየተጠቀሙበት ነው ወይ? (በምልከታ) | 1. አዎን 2. የለም | | | |  | |
| 245 | | ከተጸዳዳህ/ሽ በኋላ አፈጋራን የምታያጸዳበት/ጂበት ዘዴ የትኛው ነው? | 1. ሶፍት   2) ወረቀት   1. ውሐ 2. ቅጠል | | | |  | |
| 246 | | መጸዳጃ ቤቶቹ ሽታ የላቸውም ወይ?(በምልከታ) | 1. አዎን 2. አላቸው | | | |  | |
| 247 | | መጸዳጃ ቤቶቹ ከዝንብ ነፃ ነው ወይ? | 1. አዎን 2. አይደለም | | | |  | |
| 248 | | የትምህርት ቤቱ ግቢ ከዓይነ ምድር የጸዳ ነው ወይ? | 1. አዎን 2. አይደለም | | | |  | |
| 249 | | ውሐን ማፍላት ጀርም ይገድላል ወይ? | 1. አዎን 2. የለም | | | |  | |
| 250 | | የውሐ መያዛ ዕቃ ግጣምና መጸዳት ያስፈልገዋል ወይ? | 1. አዎን 2. የለም | | | |  | |
| 251 | | የሰው ልጅ ጽዳጅ በሽታ አምጭ ጀርም ይይዛል ወይ? | 1. አዎን 2. የለም | | | |  | |
| 252 | | እጅን መታጠብ በውሐ፣ በአካባቢ ንጽህናና በግል ንጽህና ጋር ተያይዞ በሚመጡ የሆድ በሽታዎች እንዳንያዝ ያደርጋል ወይ? | 1. አዎን 2. የለም | | | |  | |
| 253 | | እጅን ለመታጠብ ሳሙናና አመድ መጠቀም እጅ ላይ ያለን ,ጀርም ይገላል ወይ? | 1. አዎን 2. የለም | | | |  | |
| 254 | | ከውሐ፣ ከአካባቢ ንጽህናና ከግል ንጽህና ጋር ተያይዞ ስለሚመጡ የሆድ በሽታዎች ግንዛቤ አለህ/ሽ? | 1. አዎን 2. የለም | | | |  | |
| 255 | | ውሐን፣ የአካባቢ ንጽህናንና የግል ንጽህናን በአግባቡ ባለመጠበቅ ምክንያት የሚመጡ በሽታዎች አሉ ወይ ? | 1. አዎን 2. የለም | | | | የለም ወደ 257 | |
| 256 | | የተራ ቁጥር 255 መልስ ‹‹ አዎን›› ከሆነ በሽታዎቹ ምን ምን ይባላሉ? | 1. አሜባ 2. ጃርዲያ 3. የሆድ ትላትል 4. ሌላ ካለ-------------- | | | |  | |
| 257 | | የሆድ ትላትል ለማከም የሚውል ባለፈው ስድስት ወር ወስደሀል/ሻል ወይ | 1. አዎን 2. የለም | | | |  | |

ክፍል ሦስት: ከበሽታ ጋር የተያያዙ መረጃዎች

| 301 | ባለፈው አንድ ወር ውስጥ ከዓይነ ምድርህ/ሽ ጋር ትላትል ታይቷል? | 1. አዎን 2. የለም |  |
| --- | --- | --- | --- |
| 302 | ባለፈው ሁለት ሳምንት አሞህ/ሽ ነበር? | 1. አዎን 2. የለም | የለም ከሆነ ከዚህ በታች ያለውን በሙሉ |
| 303 | የተራ ቁጥር 302 መልስ ‹‹አዎን›› ከሆነ ምልክቱ ምንድን ነበር? | 1. ማቅለሽለሽ 2. ማስታወክ 3. ተቅማጥ 4. የሆድ ህመም |  |
| 304 | ከላይ የተገለጹት ምልክቶች በታዩ ወቅት ታክመሀል/ሻል? | 1. አዎን 2. የለም |  |
| 305 | በጤና ድርጅቱ ምርመራ የተለየልህ/ሽ በሽታ ምን ነበር? | 1. አሜባ 2. ጃርዲያ 3. ሌላ ካለ----- |  |

አመሰግናለሁ!
