# Supplementary material for: Water, sanitation, and hygiene conditions and prevalence of intestinal parasitosis among primary school children in Dessie City, Ethiopia
Source: PLoS One. 2021 Feb 3;16(2):e0245463. doi: 10.1371/journal.pone.0245463 (PMC7857601; doi:10.1371/journal.pone.0245463)
Supplement: S3 File — (DOCX) [file pone.0245463.s003.docx]

### Annex II. English Questionnaire

Questionnaire code___________ _

School's Name

*Kebele*________________

**Instruction -** Please encircle for the responses for each question with their alternatives and write the responses for open ended question on the space provided

**Part One: Socio-Demographic Questions**

| S. No. | Questions | Alternative/ choice of response | Skip |
| --- | --- | --- | --- |
| 101 | Age in years | ________ years |  |
| 102 | Residence | 1. Rural  2. Urban |  |
| 103 | Child's Sex | 1. Male  2. Female |  |
| 104 | Religion | 1. Christian  2. Muslim  3. Protestant  4.Other________ |  |
| 105 | Grade level | ____________ |  |
| 106 | Parental Educational status (mother) | 1. illiterate  2.Read and write  3.primary school  4.Secondary school and above |  |
| 107 | Parental Marital status (mother) | 1.Single  2.Married  3.Divorced  4.Widowed  5. died |  |
| 108 | Mother's Occupation | 1.Housewife  2.Merchant  3.Government Employee  4.Private Employee  5.Other specify |  |
| 109 | Educational status of father | 1. illiterate  2.Read and write  3.primary school  4.Secondary school and above |  |
| 110 | Father's Occupation | 1.Farmer  2.Merchant  3.Government Employee  4.Private Employee  5.Other ______________ |  |
| 111 | Household’s family size including yourself | ________________ |  |

Part Two: WaSH Factors

| Water supply factors | | | | |
| --- | --- | --- | --- | --- |
| 201 | Drinking water source at household level | 1. tap water 2. protected spring 3. protected dug well 4. others |  | |
| 202 | Drinking water source from school level (asking and observing) ( source of water for schools) | 1. tap water 2. protected spring 3. protected dug well 4. others |  | |
| 203 | Who is responsible for maintaining operation of the water sources? | 1. sub-city water and sewerage department 2. Own plumber 3. Community |  | |
| 204 | Do water treatment/disinfection performed at the school? | 1. No 2. Yes |  | |
| 205 | The capacity of water supply required per capita per day? | 1. < five litres 2. ≥ five litres |  | |
| 206 | Is there a suitable alternative supply in case of need? | 1. No 2. Yes |  | |
| 207 | Are there sufficient water points in the right places for all needs | 1. No 2. Yes |  | |
| 208 | Are there water points for disabled staff and children? | 1. No 2. Yes |  | |
| 209 | Is water accessible where needed at all times? | 1. No 2. Yes |  | |
| 210 | Are drinking-water points properly used and adequately maintained? | 1. No 2. Yes |  | |
| Hygiene factors | | | | |
| 211 | Is there health club in the school? | 1. No 2. Yes | | No Skip to 212 |
| 212 | Is hygiene and sanitation part of the health club? | 1. No 2. Yes | |  |
| 213 | Are staffs trained in providing hygiene education? | 1. No 2. Yes | |  |
| 214 | Is there responsible body for promoting hygiene in the school? | 1. No 2. Yes | |  |
| 215 | Are school facilities designed to be easily and hygienically used and maintaining | 1. No 2. Yes | |  |
| 216 | Is there hygiene education actually provided? | 1. No 2. Yes | |  |
| 217 | Do school children participate actively in maintaining hygiene? | 1. No 2. Yes | |  |
| 218 | Have the children been shown how to correctly use the toilet and water point? | 1. No 2. Yes | |  |
| 219 | Have the children been shown how to wash their hands correctly? | 1. No 2. Yes | | If no skip to Q 219 |
| 220 | If the answer for Q.219 “yes” show the hand washing steps? | 1. Yes 2. No | |  |
| 221 | Is there always soap or a suitable alternative at hand washing points? | 1. No 2. Yes | |  |
| 222 | Do staffs provide positive role models for hygiene behaviors? | 1. No 2. Yes | | No to Q 224 |
| 223 | If the answer for Q.222 is 'yes' in by what method they motivate? | 1. Maintenance lf hygiene  2. regular monitoring  3. rewarding  4. others | |  |
| 224 | Do school children clean their teeth regularly (after every meals)? | 1. No 2. Yes | | no to Q 224 |
| 225 | If the answer for number 224 “yes” what type of teeth cleaning used? | 1. water only 2. Twigs 3. Soap with water 4. Other | |  |
| 226 | Hand washing before meal | 1. Always 2. sometimes | |  |
| 227 | Hand Washing after defecation/toilet | 1. Always 2. Sometimes | |  |
| 228 | Hand washing after caring kids/babies | 1. Always 2. Sometimes | |  |
| 229 | Hand washing before food preparation or before touching utensils | 1. Always 2. Sometimes | |  |
| 230 | Hand washing After touching pet animals | 1. Always 2. Sometimes | |  |
| 231 | Hand washing after meal | 1. Always 2. Sometimes | |  |
| 232 | Dirty materials in the hands fingernails | 1. Yes 2. No | |  |
| Sanitation factors | | | | |
| 233 | Place of defecation (observation in the school) | 1. Yes 2. No | |  |
| 234 | What type of toilet available in the school? (see toilets) | 1. Latrine 2. Pour flash 3. VIP latrine | |  |
| 235 | Are there sufficient toilets (number of seats) at the school? | 1. 1 to ≤ 50 boys 2. 1 to ≤ 25 girls 3. 1 > 25 girls 4. 1 > 50 boys | |  |
| 236 | Are there separated blocks for girls and boys? (ask students and observe | 1. Yes 2. No | |  |
| 237 | Do the toilets provide privacy and security? (observe the site) | 1. Yes 2. No | |  |
| 238 | Are they appropriate and accessible for children with a disability? | 1. Yes 2. No | |  |
| 239 | Is there one accessible toilet cubicle for disabled females and one for d | 1. Yes 2. No | |  |
| 240 | Are the toilets hygienic to use and easy to clean? (ask students and observe the site ) | 1. Yes 2. No | |  |
| 241 | Are there hand washing facilities close by toilets? (observe) | 1. Yes 2. No | |  |
| 242 | Is there a cleaning and maintenance plan? (see the plan) | 1. Yes 2. No | |  |
| 243 | Are access paths of toilet kept in good condition? (observation in the school) | 1. Yes 2. No | |  |
| 244 | Are the toilets being used properly? (asking and observe the site) | 1. Yes 2. No | |  |
| 245 | Which type of anal cleansing type used by pupils? | 1. soft  2. paper  3. water  4. leaf  5. others | |  |
| 246 | Are the toilets without smell? | 1. Yes 2. No | |  |
| 247 | Are the toilets free from flies? | 1. Yes 2. No | |  |
| 248 | Are the school grounds kept free from faecal matter? (observation in the | 1. Yes 2. No | |  |
| 249 | Do you know boiling water kills germs | 1. Yes 2. No | |  |
| 250 | Do you know water container needs cleaning and covering | 1. Yes 2. No | |  |
| 251 | Do you know human feces contain germs | 1. Yes 2. No | |  |
| 252 | Do you know washing hands can prevent from infection of WaSH associated | 1. Yes 2. No | |  |
| 253 | Do you know using soap, ash for hand washing kill germs on hand | 1. Yes 2. No | |  |
| 254 | Are you aware of any water, sanitation and hygiene associated intestinal | 1. Yes 2. No | |  |
| 255 | Do you know the diseases occurred due to water, sanitation, and hygiene | 1. Yes 2. No | |  |
| 256 | If the answer for number 256 is “yes” what diseases it can be? | 1. Ameobiasis 2. Gardiasis 3. Helminthiasis 4. Others | |  |
| 257 | Have you ever been given any deworming in the last 6 months? | 1. Yes 2. No | |  |

**Part Three: Morbidity Related Data**

| 301 | Have you ever observed worms in the last one month? | 1. Yes  2. No |  |
| --- | --- | --- | --- |
| 302 | Did you get illness in the last two weeks? | 1.Yes  2. No | If no skip all part |
| 303 | If yes, what were your symptoms and signs? | 1.Nausea  2.Vomiting  3.Diarrhea  6.Adominal pain |  |
| 304 | Did you get treatment while you feel the above symptoms? | 1.Yes  2.No |  |
| 305 | Which type of diseases did you diagnose in both health institutions? | 1. Ameobiasis 2. Gardiasis 3. Others specify |  |

Thank you!
